# Outbreak investigation of *Serratia marcescens* bloodstream infection in an obstetric ward for high-risk pregnant women

**DOI:** 10.1186/s12879-024-09134-1

**Published:** 2024-02-28

**Authors:** Seulki Kim, Sunah Jung, Dong Hyung Lee, Chulhun L. Chang, Moonsuk Bae, A Reum Kim, Su Jin Lee, Seungjin Lim

**Affiliations:** 1 Department of Internal Medicine, Division of Infectious Diseases, Pusan National University Yangsan Hospital, 20 Geumo-Ro, Mulgeum-Eup, Yangsan, 50612 Republic of Korea; 2 Research Institute for Convergence of Biomedical Science and Technology, Pusan National University Yangsan Hospital, Yangsan, Republic of Korea; 3 Infection Prevention Department, Pusan National University Yangsan Hospital, Yangsan, Republic of Korea; 4 Department of Obstetrics and Gynecology, Pusan National University Yangsan Hospital, Yangsan, Republic of Korea; 5 Department of Obstetrics & Gynecology, Pusan National University School of Medicine, Busan, Republic of Korea; 6 Department of Laboratory Medicine, Pusan National University Yangsan Hospital, Yangsan, Republic of Korea; 7 Department of Internal Medicine, Pusan National University School of Medicine, Busan, Republic of Korea

**Keywords:** Infectious disease outbreaks, *Serratia marcescens*, Bloodstream infection, Whole-genome sequencing, Hand hygiene, High-risk pregnancy

## Abstract

**Background:**

*Serratia marcescens* is a gram-negative bacterium that is widespread in the environment. *S. marcescens* bacteremia can be fatal during pregnancy and cause persistent chorioamnionitis. This study reports an outbreak of *Serratia marcescens* bloodstream infection (BSI) among high-risk pregnant women in an obstetric ward. The purpose of this study is to report our experience with the usefulness of the ATP test in hospital environmental management and to confirm that bloodstream infections of patients with the same strain were correlated by WGS testing.

**Methods:**

This retrospective study collected the data of inpatients with *S. marcescens* bacteremia in obstetric ward for high-risk pregnant women from August 22, 2021, to October 14, 2021. We performed: an adenosine triphosphate (ATP) bioluminescence test in the environment with a high-contact area; environmental culture; on-site monitoring and staff education; and whole-genome sequencing (WGS) to evaluate genetic relationships among *S. marcescens* isolates.

**Results:**

*S. marcescens* BSI occurred in four consecutive patients. None of the patients had central venous catheters. An ATP bioluminescence test revealed that high-contact areas and areas for injection preparation were not clean (≥ 1000 relative light units). However, *S. marcescens* was not identified in the environmental cultures, likely due to intensive environmental cleaning and discarding of potentially contaminated specimens before the culture test. On-site monitoring and education were conducted for 1 month. There were no further reports of BSI until 6 months after the last patient was discharged. WGS performed on three isolates from three patients indicated that the isolated *S. marcescens* was likely from the same strain.

**Conclusions:**

We controlled an *S. marcescens* outbreak by improving environmental cleaning as well as education of and behavior changes in healthcare workers. Using the ATP bioluminescence test can provide feedback on environmental cleaning and education. WGS played a role in determining the spread of BSI caused by the same strain.

**Supplementary Information:**

The online version contains supplementary material available at 10.1186/s12879-024-09134-1.

## Background

*Serratia marcescens*is a gram-negative bacterium that is widespread in the environment [1, 2]. It causes severe hospital-acquired infections such as pneumonia, meningitis, bacteremia, urinary tract infections, and endocarditis [3]. Newborns admitted to neonatal intensive care units are at high risk of acquiring *S. marcescens*because of their immature immune systems [4]. Additionally, *S. marcescens*bacteremia can be fatal during pregnancy and cause persistent chorioamnionitis [5–7]. However, few studies have investigated outbreaks among pregnant women in obstetric wards [8, 9].

This report retrospectively describes the outbreak and control of *S. marcescens* bacteremia in four high-risk pregnant women from August 22, 2021, to October 14, 2021. The purpose of this study is to report our experience with the usefulness of the ATP test in hospital environmental management for outbreak control, and to confirm that bloodstream infections of patients with the same strain were correlated by WGS testing. This study followed the Outbreak Reports and Intervention Studies of Nosocomial Infection (ORION) guidelines [10].

## Methods

### Design

This report was retrospective single center study. We report our experience using ATP testing during infection outbreak control. In addition, conserved isolates from patients were analyzed by WGS to confirm that the patients' infections were related.

### Participants

**Fig. 1:**
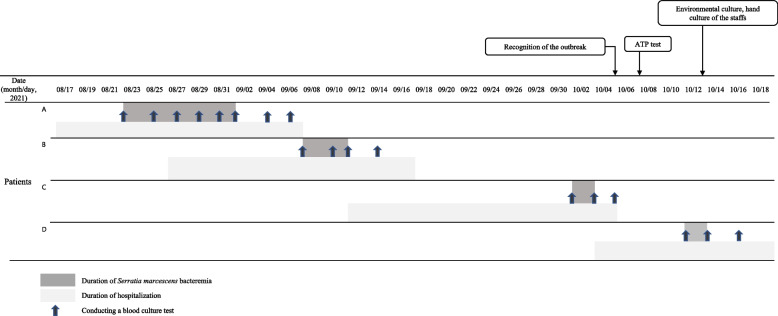
Length of hospitalization and *Serratia marcescens* bloodstream infection duration of the patients. The arrows represent each date of blood culture testing. ATP, adenosine triphosphate

Cases were defined as patients diagnosed with *S. marcescens* BSI in the ward for high-risk pregnant women between August 22 and October 13, 2021. The age range was 27–38 years, and the length of stay in the unit was 17–29 days. (Table 1). The index patient had an estimated illness onset in 22 August 2021 (Fig. 1). One additional case occurred during the outbreak investigation period. In total, four BSI cases were identified in this outbreak. *S. marcescens* BSIs that occurred in other wards during the same period were excluded if there was no movement of patients or healthcare personnel.

**Table 1:** Clinical outcomes of the patients with *S. marcescens* blood stream infection

	Patient A	Patient B	Patient C	Patient D
Age (years)	27	34	38	35
GA at the admission (weeks + days)	27 + 1	21 + 2 (twin)	33 + 5	29 + 4 (twin)
Major diagnosis	Preterm labor	IIOC and Preterm labor	Preeclampsia	TTTS and Preterm labor
Treatment before BSI	Tocolytics (Atosiban + magnesium)	Tocolytics (Ritodrine + magnesium)	Tocolytics (magnesium)	Tocolytics (Ritodrine + Atosiban + magnesium)
Start date of BSI during hospitalization	HD 5	HD 13	HD 21	HD 8
Length of stay in the unit (days)	25	21	29	17
Symptom of BSI	Fever	Fever	Fever	Fever
Prognosis of patients	Unknown^a^	Recovery	Recovery	Recovery
Prognosis of fetus	Unknown^a^	No evidence of *S. marcescens* infection	No evidence of *S. marcescens* infection	No evidence of *S. marcescens* infection
Preterm birth (GA, weeks + days)	Unknown^a^	Yes (36 + 1)	Yes (36 + 5)	Yes (31 + 3)
Association between BSI and preterm birth	N/A	No	Yes^b^	Unknown^c^
Transmission of *S. marcescens*	No	No	No	No

*Abbreviations*: *IIOC* Incompetent internal os of cervis, *TTTS* Twin to twin transfusion syndrome, *BSI* blood stream infection, *HD* hospital day, *GA* gestational age, *ICT* immunochromatography-based rapid diagnostic tests

^a^Patient A was transferred to another hospital; thus, her obstetric outcomes could not be identified

^b^Patient C underwent an emergency cesarean section the day after developing *S. marcescens* BSI. After the onset of fever, the surgery was performed due to the aggravation of preeclampsia, and it is considered to be related

^c^After resolving bacteremia, patient D underwent an emergency cesarean section due to symptomes like uterine contractions and leaking amniotic fluid

**Fig. 2:**
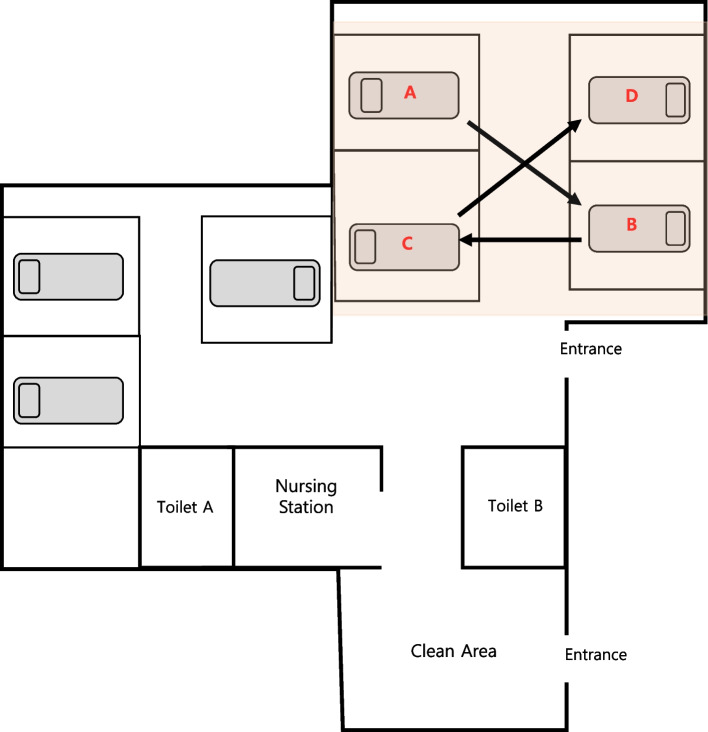
Floor plan of the ward and the order of infected patient occurrence. The onset of the outbreak occurred in sequential order, starting from the initial patient A (index case) and progressing to subsequent patients (B, C, D). Arrows: the order and path of infection propagation

The four patients were hospitalized between August 12 and October 18, 2021. They developed fever at least 5 days after hospitalization (within a range of 5 to 21 days), and *S. marcescens* was confirmed in the blood culture taken within 2 h of fever onset. (Table 1) A total of 47 patients were hospitalized in the same ward during this period. Of the two sections of the ward, the outbreak occurred in only one section of the ward. A total of 26 patients were hospitalized in the outbreak district area (Fig. 2).

### Setting

Pusan University Yangsan Hospital has 1,205 beds and is a teaching tertiary hospital with 42,000 admissions, accounting for 309,000 patient-days per year. The infection control department was staffed by nine nurses and two infectious disease physicians in 2021. The obstetric ward has seven beds for the care of pregnant women at risk for premature birth, such as those with gestational hypertension, premature contraction of the uterus, premature rupture of membranes, and premature labor.

The ward is divided into two sections composed of three and four beds (Fig. 2). The outbreak was limited to one district. An average of 18 patients are hospitalized per month in the ward (1,942 patient-days in the ward per year). One doctor was on duty from 9:00 a.m. to 6:00 p.m. and was supervised by a faculty member. An on-call doctor was available during non-business hours. Nurses were working three shifts, with one nurse assigned to each shift.

The patient population in the obstetric ward for high-risk pregnant women differs from that in other general adult wards in our hospital. First, the population is limited to pregnant women. Second, none of the patients had received long-term health care treatment prior to their pregnancy-related illness. Third, the frequency of central lines and indwelling catheters is very low, and ventilators are not used in the ward. From 2018 to June 2022, the mean monthly incidence of BSI was 0.77% among total hospitalized patients in this ward. In contrast, during the BSI rate during the *S. marcescens* outbreak alone was 8.51%. No infections from multidrug resistant *Acinetobacter baumannii* or *Pseudomonas aeruginosa* and Carbapenem-resistant *Enterocacteriaceae* were reported during this period. (Supplementary Material 1).

Hospitalized pregnant women in this ward received intravenous injections and necessary tests depending on the patient’s condition. Most injections were administered using peripheral veins. Atosiban, magnesium sulfate, or ritodrine were the most used injectables in the ward. In routine practice, non-stress tests (NST) were conducted twice a day, and ultrasounds were performed once every 1–2 weeks. If the patient’s cervical length was short, transvaginal ultrasounds were performed approximately twice a week.

### Outbreak recognition

On October 5, 2021, the infection prevention department of the study center received a report regarding three consecutive cases of *S. marcescens* bloodstream infection (BSI) in the obstetric ward. Since 2015, there have been no reports of *S. marcescens* outbreak in the hospital. No other outbreaks caused by other bacteria in the obstetric ward have been confirmed.

After recognizing the outbreak, the clinical microbiology department requested the preservation of available *S. marcescens* isolates from the patients to perform WGS. Additionally, an on-site investigation was planned in consultation with the ward medical staff.

### Interventions

#### Investigation of the environment and staff

On October 7, 2021, the nurse from the infection prevention department visited the ward to perform an on-site inspection and ATP bioluminescence test (3 M™ Clean-Trace™ Surface ATP; 3 M, St. Paul, MN, USA) [11]. The test was performed focusing on high-contact areas (Table 2). We considered the threshold for repeat cleaning to be an ATP bioluminescence level of 250 relative light units (RLU)/100 cm^2^and further defined ≥ 1000 RLU as “not clean.” [11, 12].

**Table 2:** ATP bioluminescence measurements for outbreak investigation

Inspection area	ATP bioluminescence (RLU)	Need for additional cleaning (> 250 RLU)	Not clean (> 1000 RLU)
C bedside rail	425	+	-
C bed monitoring system	545	+	-
C bed blood pressure cuff	2694	+	+
C bed’s closet	322	+	-
C bed’s call button	705	+	-
NST of C bed	175	-	-
C bed pad for NST	81	-	-
Handrail of the injection pole	467	+	-
Tray for injection	2668	+	+
UDS cart in the ward	90	-	-
Computer mouse #1	394	+	-
Computer keyboard #1	503	+	-
Call button of nursing station	174	-	-
Computer mouse #2	365	+	-
Computer keyboard #2	4515	+	+
Doppler device	674	+	-
Touchpad of door	1901	+	+
Infusion pump before use	1002	+	+
Tray for injection in the delivery room	2471	+	+
UDS cart in the delivery room	210	-	-
Probes for transvaginal sonography in the obstetric ward	396	+	-
Button #1 of ultrasonography	80	-	-
Button #2 of ultrasonography	192	-	-
Probes for transvaginal ultrasonography in the delivery room	61	-	-
Total (*N* = 24)		16	6

*Abbreviations*: *ATP* adenosine triphosphate bioluminescence, *NST* non-stress test monitor, *RLU* relative light units, *UDS* unit dosage system

Hand hygiene monitoring of healthcare workers was conducted from October 7 to October 12, 2021. Six days later, on October 13, 2021, an environmental culture test was performed. Environmental surface samples were homogenized in a stomacher, and eluents were cultured on tryptic soy agar plates containing 5% sheep blood and MacConkey II plates. Sampling was performed using sterile swabs inserted into plastic tubes. Environmental cultures were conducted for the products or surfaces of the patient and healthcare personnel areas (Table 3). Additionally, culture samples were obtained from the staff’s (three nurses and two doctors) hands.

**Table 3:** Products and surfaces tested in the environmental culture

	Inspection area	Number of obtained specimens for culture
Common area	Touchpad on automatic door	1
Patient’s area	Handle of a shared refrigerator	1
	Blood pressure cuff	1
	Side rails of beds	1
	Tables in beds	1
	Patient monitoring system	1
	Call button on the bed	1
	Gel for ultrasonography beside the bed	4
	Hand sanitizer beside the bed	4
	Faucet	2
	Toilet seat lid	2
	Toilet button surface	2
	Faucet on the bidet	2
	Water in the bidet	2
	Bidet dryer nozzle	2
	Basin surface	2
	Bathroom scales	2
Healthcare personnel’s area	Sink surface	1
	Faucet	1
	Water soap	1
	Computer keyboard table	1
	Computer mouse surface	1
	Infusion pump surface	1
	Environmental disinfectants	1
	Surface above the unit dosage system cart	1
	Injection tray	1
	Probe on an ultrasonography	1
Testing healthcare workers’ hand	Nurse	3
	Doctor	2
Total^a^		46

^a^None of the environmental cultures identified *Serratia marcescens*

#### Education and feedback of the staff through on-site monitoring and self-report

From October 14, 2021 to November 14, 2021, hand hygiene monitoring and on-site education were conducted by an infection control nurse once a day, Monday through Friday, at a set time.

We checked whether the obstetrical ward staff complied with the following items and provided feedback on the spot: seven steps and five points of hand hygiene [13], safe injection practices [14], guidelines for cleaning the probe for transvaginal sonography [15], and guidelines for environmental cleaning management [16]. The ward also changed its staffing. Changed from one nurse to two nurses per shift. Moreover, ward staff were asked for their opinions on obstacles to complying with the ward’s standard precautions at the meeting for outbreak control. After the on-site training period, hand hygiene monitoring was conducted again from November 15 to November 19, 2021.

### Culturing and typing

#### Antibiotic susceptibility testing

Identification of isolates to the species level was achieved using Matrix-Assisted Laser Desorption Ionisation Time of Flight Mass Spectrometry (MALDI-TOF MS; bioMerieux, Nurtingen, Germany), and antibiotic susceptibility testing was performed using VITEK-2 (bioMerieux, Durhum, NC, USA). The results were interpreted according to the identification criteria for *S. marcescens*, using the Clinical and Laboratory Standards Institute guidelines [17].

#### WGS

Isolates from the index case were discarded when the outbreak was recognized. Therefore, WGS was performed on the isolates from three patients. The genomes of *S. marcescens* isolates were fully sequenced using Illumina MiSeq (Illumina, Inc., San Diego, CA, USA). Phylogenetic tree inference was performed on the set of genomes, including sample genomes obtained in this study, type strains of *Serratia* (*n* = 18), and representative genomes of the dereplicated clusters of genomes that had a whole-genome Mash distance < 0.1 with one or more of the sample genomes. The core genes of *Serratia* were predefined from the available genomes of *Serratia*spp. in the CJ Bioscience bacterial genome database using CoreCruncher [18]. The presence of closely related strain pairs within or between the samples and the reference genomes was first screened using an in-house *Serratia*core genome multi-locus sequence typing (cgMLST) scheme built with 868 core genes [19]. We analyzed core genome single nucleotide polymorphisms (cgSNPs) to provide more reliable clone-level relatedness among genomes. The SNPs were counted in the core genomes of the samples and reference genomes using split kmer analysis version 1.0 [20]. We included pairwise cgSNP distances for the set of strains that included the sample strains and any strain showing <${1\times {\text{e}}}^{-4}$SNP distances with any of the sample strains. If the three identified isolates belonged differently to several clusters or if the SNP difference was too significant compared to the time scale in which transmission could occur, it was regarded as individually acquired rather than transmission between patients. To screen for potential transmission caused by pathogen, we defined isolate pairs with ≤ 7 to 10 core SNP differences [21].

#### Infection-related outcomes

Patient C underwent an emergency cesarean section the day after developing *S. marcescens* BSI. No patient died from *S. marcescens* bacteremia, and no neonate born to the patients showed evidence of *S. marcescens* infection (Table 1). Patient A was transferred to another hospital; thus, her obstetric outcomes could not be identified.

#### Potential threats to internal validity

There was no change in antibiotic policies compared to the same period one year prior to the outbreak. The ward's hand hygiene performance rate was maintained at around 85%-90% until June 2021. There were no significant changes of hand hygiene before the outbreak.

## Results

### Cluster descriptions

#### Epidemiological outbreak investigation

During the outbreak, 47 high-risk pregnant women were hospitalized in the ward. The outbreak occurred in only one unit (Fig. 2), where 26 patients were hospitalized. Four women were diagnosed with bacteremia caused by *S. marcescens* during the outbreak period (Table 1). Between August 22 and October 3, 2021, three patients (A, B, and C) developed *S. marcescens* BSI. Bacteremia also occurred in one other patient (D) on October 11, 2021, during the investigation period. No other pathogenic infections were identified among hospitalized patients during the outbreak.

Patient A was considered the index case. *S. marcescens* is shown in Fig. 1. The hospitalization period and BSI duration for each patient are presented in Fig. 2. When the outbreak was recognized, there were no remaining samples for the index case. It had been discarded after a four-week storage period. Except for the index case, the microorganisms isolated from the three patients were preserved in the clinical microbiology department. No bacteria were identified in the urine samples.

#### ATP bioluminescence test and environmental culture

The ATP bioluminescence test was performed in 24 environments by multiple medical staff members (Table 1). Sixteen locations required recleaning (> 250 RLU) [12]. Six locations were not clean (> 1000 RLU) [12]. The infusion pump and injection tray related to injection administration were not clean even before use. High ATP bioluminescence test results (> 1000 RLU) were detected in the patients’ blood pressure cuff, on the computer keyboard in the staff area, and on the touchpad of the door in the ward, with a high-contact area.

Forty-one environmental samples were obtained from these cultures (Table 2). Cultures were also performed on the hand sanitizer, ultrasound gel, and hands of staff. However, *S. marcescens* was not identified. The gel used in the outbreak investigation was discarded because it was improperly stored with the lid open; therefore, the test could not be performed on the previously used gel. *S. marcescens* was not identified in the hand culture of the ward staff.

We observed two issues during environmental culture testing: 1) intensive environmental cleaning was performed before the environmental culture tests (based on the results of the ATP bioluminescence test), and 2) the ultrasound gel that needed to be cultured was discarded before the test. These things might have affected the culture test results.

#### Recognizing and solving the problems: on-site monitoring, education, and meetings with staff in charge

While investigating the cause, the following problems were observed during the on-site investigation: the lack of hand hygiene among the medical staff; inadequate injection safety (preparing injections in unclean areas, lack of hand hygiene, scrubbing the injection port); failure to comply with the guidelines’ disinfection time for the probe for transvaginal sonography [2]; ultrasound gel stored in an open state and against the manufacturer's recommendations; and daily environmental cleaning management not in accordance with the guidelines of the study center due to a lack of human resources.

#### Implementation of infection control measures

First, on-site education of medical staff was attempted to correct the lack of hand hygiene and safe injection practices. Second, a new disinfectant (Tristel™ DUO; Tristel, Snailwell, UK) that requires a shorter time than the one previously used (CIDEX™ OPA; Advanced Sterilization Products, Irvine, CA, USA) was introduced to comply with the disinfection time for transvaginal sonography probes. Third, cleaning the ward was made a priority for the healthcare personnel in charge. The cleaning personnel were then redeployed. Nurses also cleaned high-contact areas, such as computers, keyboards, telephones, and desks, before starting work. They utilized disinfecting wipes containing quaternary ammonium compounds (MD125 wipes; Jun Medicare, Korea), which are the disinfectant wipes used to disinfect surface environments in the hospital. Fourth, healthcare personnel were trained in the proper storage method for the ultrasound gel. Accordingly, monitoring and feedback were conducted once a day, except on weekends, by the infection prevention department. No additional cases occurred during the 6 months after the last patient (D) was discharged. Changes in hand hygiene performance from pre- to post-infection control interventions are shown in Table 4. Prior to the outbreak, routine monitoring showed hand hygiene performance at 85–90%. However, during ward rounds after the outbreak was recognized, we noticed a further decline in performance. After a month of on-site feedback and training, we saw an improvement in hand hygiene performance, from 55.1% to 85.5%.

**Table 4:** Changes in hand hygiene performance from pre- to post-infection control intervention

	Pre-intervention period		Post-intervention period	
	Performed/Observed count	Performance rate	Performed/Observed count	Performance rate
Nurses	37/61	60.0%	40/47	85.1%
Doctors	1/8	12.5%	31/36	86.1%
Total	38/69	55.1%	71/83	85.5%

### Investigation using antibiotic susceptibility and WGS tests

**Fig. 3:**
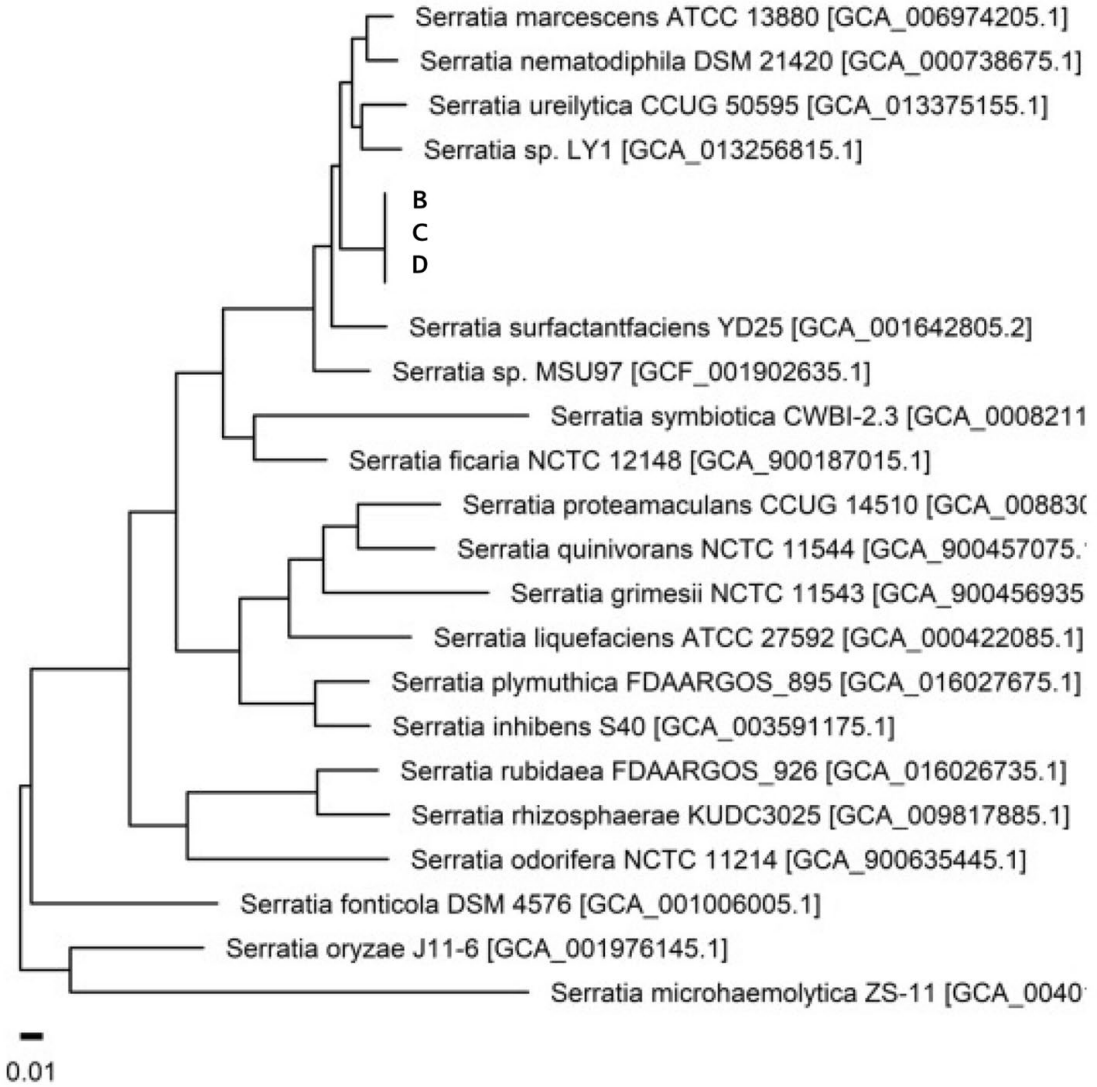
Phylogenetic tree of the samples based on the alignments for 868 core genes of the genus *Serratia*

The antibiotic susceptibility results of *S. marcescens* identified in the four patients were similar (Table 5). WGS was performed on the three strains for which the specimens were preserved. These factors were linked during the outbreak. Figure 3 shows the core gene phylogeny of the samples and the type strains of *Serratia*. Figure 3 suggests that the three samples belong to the same strain.

**Table 5:** Antibiotic susceptibility of *Serratia marcescens* isolates during the outbreak

Antibiotics	MICs (μg/mL)	Interpretation
Amoxicillin/clavulanate	≥ 32	resistant
Amikacin	≤ 2	sensitive
Aztreonam	≤ 1	sensitive
Ceftazidime	≤ 1	sensitive
Cefazolin	≥ 64	resistant
Ciprofloxacin	≤ 0.25	sensitive
Cefotaxime	≤ 1	sensitive
Ertapenem	≤ 0.5	sensitive
Cefepime	≤ 1	sensitive
Gentamicin	≤ 1	sensitive
Imipenem	≤ 0.25	sensitive
Trimethoprim/Sulfamethoxazole	≤ 20	sensitive
Tigecycline	2	sensitive
Piperacillin/Tazobactam	≤ 4	sensitive

Minimal inhibitory concentration was interpreted according to the Clinical and Laboratory Standards Institute (CLSI) guidelines. Interpretation categories include 'resistant’, 'intermediate’, or 'sensitive’. MICs; Minimal inhibitory concentrations

Additionally, the distribution of pairwise cgSNP distances was calculated from the genomes of *Serratia*, including both sample and reference strains. All three isolates were significantly related to each other (Table 6). Two samples from patients C and D were more closely related, whereas the sample from patient B was relatively less correlated with the other two samples in the sequencing of antibiotic resistance proteins. However, all three were close enough to be considered causative of the same outbreak in the WGS analysis (cutoff ≤ 7 to 10 core SNP differences) [21]. The cgSNP distanace between the 3 strains was: ${2\times {\text{e}}}^{-6}$ (SNP sites per total core genome sites) and the maximum number of cgSNPs was no more than 2.

**Table 6:** Core genome single nucleotide polymorphism (cgSNPs) distance between samples

Sample 1	Sample 2	Number of cgSNPs	cgSNP distance
B	C	2	${2.2\times e}^{-6}$
B	D	2	${2.2\times e}^{-6}$
C	D	0	0

## Discussion

The outbreak of *S. marcescens* in obstetric wards has important implications because it can affect the colonization of *S. marcescens* in newborns and lead to chorioamnionitis caused by *S. marcescens* [9, 22]. This study investigated the outbreak of *S. marcescens* BSIs among hospitalized pregnant women on four adjacent beds.

Hospital-acquired BSIs caused by *S. marcescens* were observed in patients without central venous catheters. In 1993, a study reported an outbreak in which 17 obstetric patients and 11 concurrent neonates were infected with *S. marcescens*; the source of the outbreak was contaminated gel used during pelvic examinations [8]. Another study in 1999 reported an outbreak that ended after removing the contaminated transducers of internal tocographs [9]. These studies suggest the need for proper infection control in the delivery room to prevent pathogen colonization in newborns [8, 9].

Although our study could not prove the source of infection, the transmission of *S. marcescens* BSI was presumed to occur through hand contact among healthcare workers. The unclean conditions identified in the ATP bioluminescence test, including the injection preparation area, as well as the reduced hand hygiene practices and lack of adherence to safe injection practices, might have been responsible for this outbreak. ATP bioluminescence tests are used to measure the levels of organic residue [23, 24] and can be a valuable tool for measuring the efficiency of cleaning procedures [11]. Owing to their ease of execution and the immediacy of results, they allow surfaces to be monitored more frequently and in greater numbers. They can also be used as a rapid tool for screening the efficacy of cleaning procedures [11]. This study suggests that on-site monitoring of compliance with standard precautions and ongoing environmental management of contamination-prone areas played a role in ending the outbreak. Furthermore, ATP bioluminescence test results helped improve healthcare providers' awareness and behavior.

WGS enables detailed typing of microorganisms with higher resolution than older techniques and is the current standard method in outbreak investigations for most bacterial species [4, 23]. This study described an outbreak investigation of *S. marcescens* in the obstetric ward, analyzed using WGS, cgMLST, and cgSNP. We believe that WGS was necessary to investigate the *S. marcescens* outbreak in high-risk pregnant women. WGS revealed that the BSIs with variable onsets were related to each other except for the index case, in which the sample was not preserved. This finding could be evidence that the approximately 3 months outbreak was due to environmental problems: first, failure to comply with disinfection time guidelines for transvaginal sonography probes; second, inappropriate storage of ultrasound gel with the lid open, contrary to the manufacturer's recommendations; and third, inadequate daily environmental cleaning management, primarily attributed to a shortage of human resources.

However, the specific source of the infection was not identified. Some studies also failed to report a clear sources [25, 26], although others have identified sources of *S. marcescens*infection (ultrasound gel, razors, milk, disinfectant, bottles of enteral feed, creams used during pelvic examinations, transducers of internal tocographs, and transesophageal echocardiography probes) [3, 8, 9, 23, 27]. Recently, a study has reported using metagenome analysis to identify resistant strains in hospital environmental samples [28]. The availability of these technologies in outbreak investigations would further assist in identifying the source of infection in hospital environments.

This study had several limitations. First, the isolate of *S. marcescens* could not be preserved because the outbreak was recognized after the specimen from the index case was discarded. Second, due to a lack of human resources, ATP bioluminescence testing and environmental culture could not be performed simultaneously. Third, some samples that were likely sources of infection were discarded before environmental culture. Fourth, no molecular methods were applied for pathogen detection in the environmental samples. Finally, we could not screen all hospitalized patients and staff for colonization. 

## Conclusions

The *S. marcescens* BSI outbreak was successfully terminated through the implementation of comprehensive environmental management measures. This included the intensification of cleaning and disinfection protocols, involving an increased frequency and thoroughness of cleaning procedures. Concurrently, standard precautions were reinforced to further enhance infection control within the healthcare setting. WGS analysis may be an essential tool for confirming hospital-acquired infections among patients. Metagenome analysis of environmental specimens can also be helpful in future outbreak investigations. The ATP bioluminescence test was used to monitor and provide feedback on hospital environmental management during the outbreak. Compliance with strict standard precautions for healthcare workers is essential to prevent the spread of *S. marcescens*.

### Supplementary Information

**Supplementary Material 1.**

## Data Availability

The data used in this study are available from the corresponding author on reasonable request.

## Abbreviations

ATP: Adenosine triphosphate
BSI: Bloodstream infection
cgMLST: Core genome multi-locus sequence typing
cgSNP: Core genome single nucleotide polymorphism
SNP: Single nucleotide polymorphism
WGS: Whole-genome sequencing
