# Supplementary material for: Outbreak investigation of Serratia marcescens bloodstream infection in an obstetric ward for high-risk pregnant women
Source: BMC Infect Dis. 2024 Feb 28;24:266. doi: 10.1186/s12879-024-09134-1 (PMC10900556; doi:10.1186/s12879-024-09134-1)
Supplement: Supplementary file 1 — Supplementary Material 1. [file 12879_2024_9134_MOESM1_ESM.pdf]

Additional file 1. Results of microbial culture and identification of clinical specimens from 2018 to 2022

Specimen	Identified microorganism (n = number of patients)
Blood	*Serratia marcescens* (6)<br>4: number of patients related to the outbreak<br>2*: number of patients unrelated to the outbreak<br>*Escherichia coli* (1)
Urine	*Enterococcus faecalis* (1)<br>*Klebsiella pneumonia* (1)
Cervicovaginal swab	*E. coli* (2)<br>*Streptococcus anginosus* (1)<br>*E. faecalis* (2)<br>Gram (+) non-spore-forming bacteria (1)<br>*Lactobacillus* spp. (3)<br>*Candida* spp. (5)<br>*Staphlococcus epidermidis* (1)

*There was one patient each in 2019 and 2022. The infection was presumed to be hospital-acquired and no additional patients with bacteremia were observed.
